# Upregulation of p16^*INK4A*^ and Bax in p53 wild/p53-overexpressing crypts in ulcerative colitis-associated tumours

**DOI:** 10.1038/sj.bjc.6602050

**Published:** 2004-08-03

**Authors:** T Yoshida, N Matsumoto, T Mikami, I Okayasu

**Affiliations:** 1 Department of Pathology, Kitasato University School of Medicine, 1-15-1, Kitasato, Sagamihara, Kanagawa 228-8555, Japan

**Keywords:** ulcerative colitis, dysplasia, p53 gene mutation, p53 overexpression

## Abstract

In ulcerative colitis (UC)-associated tumours, p53 gene mutations and p53 protein overexpression are frequently found in early stages, but the two types of alteration do not always coincide. To clarify this discrepancy, p53 mutations and expression of p53-associated molecules were analysed in UC-associated dysplasias by a combination of microdissection, polymerase chain reaction-direct sequencing and immunohistochemistry at the single crypt level. Mismatch of p53 protein overexpression (+)/mutation (−) or p53 overexpression (−)/gene mutation (+) was found in nine crypts in regenerative mucosa (19 crypts), in 27 in low-grade dysplasia (41), in one in high-grade dysplasia (5) and in 12 in invasive carcinomas (17). Regarding these mismatched crypts of the first type, significant increase in p16^*INK4A*^ and Bax expression was found. The Ki-67 labelling index was depressed in such p53-diffusely positive lesions with the wild-type p53 gene, compared to their p53-diffusely positive and mutant type counterparts. p16^*INK4A*^ was upregulated indirectly as part of the negative feedback, and increase in Bax, directly controlled by wild-type p53, indicates upregulation of apoptosis. No significant relation with p53-related gene products was detected with the p53 protein overexpression (−)/p53 mutation (+) mismatch. Therefore, a tumorigenesis pathway independent of p53 dysfunction appears to exist in association with ulcerative colitis.

With longstanding ulcerative colitis (UC), dysplasias and carcinomas develop frequently, so that clinical surveillance of affected individuals by periodic endoscopic screening and random biopsy of colorectal mucosa is advisable (Blackstone *et al*, 1981; Collins *et al*, 1987; Lennard-Jones *et al*, 1990). Compared to the sporadic carcinoma case, p53 gene mutations are more frequent in early stages of tumorigenesis, being found in low-grade dysplasia (LGD) or non-neoplastic regenerative mucosa with ulcerative colitis (Chaubert *et al*, 1994; Holzmann *et al*, 1998; Takaku *et al*, 2001). Dysplasias and carcinomas associated with UC tend to develop as multiple and superficially extended lesions called DALM (dysplasia-associated lesion or mass) (Blackstone *et al*, 1981; Collins *et al*, 1987; Lennard-Jones *et al*, 1990; Okayasu *et al*, 1993). Furthermore, DALMs appear to be frequent in areas of more active inflammation. Therefore, a chronic inflammation – dysplasia – carcinoma sequence has been proposed.

Alterations of the p53 gene, frequent in late-stage sporadic colorectal carcinomas (Fearon and Vogelstein, 1990), have been reported in UC-based dysplasia, as analysed in terms of genetic instability (Willenbucher *et al*, 1999), chromosomal alterations (Willenbucher *et al*, 1997; Aust *et al*, 2000) and polymerase chain reaction (PCR)-single strand conformational polymorphism (SSCP) (Yin *et al*, 1993). Particularly, p53 mutations have been reported to be early events detected by indirect methods, such as loss of heterozygosity (LOH) (Brentnall *et al*, 1994) and PCR-SSCP (Holzmann *et al*, 1998). As it is important to identify single crypt level mutations to understand UC-associated tumorigenesis, we surveyed the whole coding region of p53 for mutations of single crypts within lesions, using our novel method of microdissection from serial histologic sections (Yoshida *et al*, 2003). We found p53 gene mutations in LGD and even in regenerative crypts of longstanding UC as well as high-grade dysplasia (HGD) and invasive carcinoma, but they did not always coincide with protein overexpression. Therefore, to clarify mechanisms underlying this discrepancy, mutations and p53-related, and expression of, cell cycle-related gene products (p53, p21^*Cip1*^, p27^*Kip1*^, p16^*INK4A*^, Mdm2, p14^*ARF*^, Bax, Ki-67) were analysed at the single crypt level in UC-associated dysplasia and carcinomas by a novel combination of microdissection, PCR-direct sequencing and immunohistochemistry (IHC). The results provided evidence of a novel pathway for UC-associated tumorigenesis, involving upregulation of p16^*INK4A*^ and Bax, which may be independent of p53 dysfunction.

## MATERIALS AND METHODS

### Histopathological Analysis

In total, 10 cases of long-term UC (21–64 years old, duration of 6–20 years) were selected. For comparison, 13 sporadic colorectal adenocarcinoma cases, excluding hereditary nonpolyposis colorectal carcinomas, were chosen, and cancer lesions and samples of normal mucosa at least 2 cm distant were subjected to analysis.

### Microdissection and full-length p53 mutation analysis of single crypts

**Figure 1:**
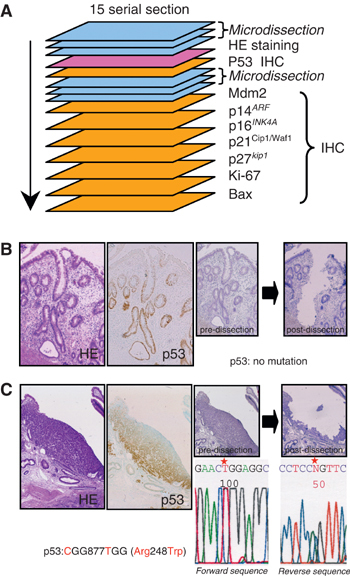
(**A**) The strategy for the microdissection, IHC and PCR-direct sequencing. After mapping of lesions by microscopic observation, 15 serial sections were prepared from each block. The fourth section in each series was stained for HE, and the fifth was devoted to p53 IHC. Sections from the first to third and sixth to eighth were used for microdissection and PCR amplification of the entire coding region of p53. Remaining sections were used for IHC of Mdm2, p14^*ARF*^, p16^*INK4A*^, p21^*Cip1/Waf1*^, p27^*Kip1*^, Ki-67 and Bax, respectively. (**B**,**C**) Examples of LGD crypts (**B**) and UC-Ca lesions (**C**) selected for microdissection. Serial sections with HE and p53 immunostaining are shown on the left-hand side. Meyer's hematoxylin staining before and after microdissection in same sections are shown on the right hand side. No mutations of p53 were found in (**B**). Mutation in codon 248 was found in (**C**) shown in partial electrophoregrams of forward and reverse sequencing of p53 exon 7. Red stars indicate the mutated nucleotides.

Serial deparaffinised sections of formalin-fixed and paraffin-embedded materials for microdissection, hematoxylin–eosin (HE) staining and p53 immunostaining were prepared as described previously (Yoshida *et al*, 2003). Briefly, from a total of 15 serial 4 *μ*M-thick histologic sections, five were applied for microdissection and the remainder for HE and IHC (Figure 1

). Tissues from single crypts were microdissected manually, and DNA was extracted by a conventional method. The whole coding region of p53 (exons 2–11) was amplified by nested PCR, and DNA sequences were analysed in forward and reverse directions with the aid of an auto sequencer (ABI310 Genetic Analyser, PE Applied Biosystems, Foster City, CA, USA).

### Antibodies

Anti-p53 (DO-7; Novocastras Lab. Ltd, Newcastle, UK; diluted × 300), anti-p27^*Kip−1*^ (Transduction Laboratory, Lexington, Kentucky, USA; × 300), anti-p21^*Cip1*^ (Oncogene Research Products, Boston, MA, USA; × 300), anti-p16^*INK4A*^ (C-20, Santa Cruz Biotechnology Inc., Santa Cruz, CA, USA; × 250), anti-p14^*ARF*^ (FL-132, Santa Cruz Biotechnology; × 200), anti-Mdm2 (SMP 14, DAKO, Copenhagen, Denmark; × 500), anti-Bax (Transduction Laboratories; × 300) and anti-Ki-67 antibodies (MIB-1, DAKO; × 300) were applied for IHC.

### Immunohistochemistry

Immunohistochemistry was performed using a combination of the standard streptoavidin–biotin–peroxidase complex (LSAB2 system, DAKO, Copenhagen, Denmark) method with antigen retrieval, as described in our previous report (Yoshida *et al*, 2003). Faint nuclear counterstaining was achieved with methyl green. Immunopositivity for p53 in crypts was defined as: positive staining of the whole crypt, ++; positive staining of the lower half of the crypt, +; scattered, ±; and negative, −, modifying the previously described method (Baas *et al*, 1994). Immunopositive cells for all markers (labelling indices) were counted or scored for each individual crypt. Scoring of Bax expression was performed by multiplying the intensity (0–3) and area (0–3). Positivity for other molecules was calculated as percentages of positive cells out of 1000 cells.

Statistical significances were analysed by Fisher's PLSD test, utilising StatView software (version 5.0).

### Western blotting

For confirmation of IHC, Western blotting of Bax and p16^*INK4A*^ was performed for representative lesions (sporadic colon cancers and normal colonic mucosa, seven cases; UC-associated invasive carcinomas and regenerative mucosa, two cases). Aliquots of 100 mg of normal mucosal and carcinoma tissue were lysed in 30 *μ*l of phospholysis buffer (Han *et al*, 1996). In total, 30 *μ*g of total cell lysate were subjected to 15% SDS–PAGE, and transferred to Hybond-P, PVDF membranes (Amersham Pharmacia Biotech, Little Chalfont, Buckinghamshire, UK). Immunoblotting was carried out with the ECL chemiluminescence system (Amersham Pharmacia Biotech) with 500 × diluted anti-p16^*INK4A*^, 750 × diluted anti-Bax and 5000 × diluted anti-*β*-actin antibodies (AC-15, Sigma, Saint Louis, MO, USA) as the primary, and anti-rabbit immunoglobulin–HRP (Amersham Pharmacia Biotech), or anti-mouse immunoglobulin-HRP (DAKO) as the secondary antibodies.

### Reverse transcription–quantitative PCR

Real-time quantitative PCR analysis for p16^*INK4A*^ was performed using the ABI PRISM 7700 Sequence Detection System and software (PE Applied Biosystems, Inc., Foster City, CA, USA). Total RNA from specimens of UC-associated carcinomas and nontumour mucosa were extracted with TRIzol reagent (Invitrogen). cDNA was synthesised from 3 *μ*g of total RNA with TaqMan Reverse Transcription Reagents (PE Applied Biosystems). p16^*INK4A*^ and primer and probe set were purchased (Pre-Developoed TaqMan Assay Reagents; PE Applied Biosystems, Cat.No. 4319445 T). Glyceraldehyde-3-phosphate dehydrogenase (GAPDH) was used as endogenous control to standardise for the amount of RNA in each reaction (TaqMan GAPDH control reagents; PE Applied Biosystems). cDNA was amplified using the TaqMan Universal PCR Master Mix (PE Applied Biosystems) according to the manufacturer's instructions on an ABI PRIM 7700 Sequence Detector. Each reaction was performed in a 25 *μ*l volume containing 2 *μ*l out of 20 *μ*l of reverse transcription reaction mixture. The cycling conditions were 50°C for 2 min and 95°C for 10 min, followed by 50 cycles of 95°C for 15 s, and 60°C for 1 min. All samples were amplified in triplicate. Copies (10–10^8^) of GAPDH plasmid were amplified to generate standard curves to relate threshold to log input amount of template. The standard curve of the GAPDH amplification was obtained as *y*=(slope) log *x*+(*Y*-intercept). The threshold cycles (*C*_T_'s) for GAPDH and p16^*INK4A*^ of nontumour mucosa (N) and UC carcinoma (T) were calculated. From the standard curve, amounts of DNA at the threshold cycle were calculated as (cDNA)=(cDNA_0_)(1+*e*)^C_T_^ (e; amplification efficiency=10^−1/(slope)^−1), and the cDNA ratio of tumour against normal tissue was calculated as follows: *R*_T/N_=(1+*e*)*C*_T_^T^/(1+*e*)*C*_T_^N^.

## RESULTS

### p53 mutations and p53 overexpression (Table 1)

**Table 1:** Relation of p53 mutation to p53 protein overexpression in single crypts

**Histology**	**crypts^a^**	**p53 mutations**	**p53 protein accumulations (wild/mutant)^b^**			
			−	**+/−**	+	**++**
UC						
REG	19	8/19 (42.1%)	14 (8/6)	1 (0/1)	1 (0/1)	3 (3/0)
LGD	41	14/41 (38.1%)	16 (8/8)	2 (1/1)	8 (7/1)	15 (11/4)
HGD	5	5/5 (100%)	1 (0/1)	0	1 (0/1)	3 (0/3)
Inv. Ca	7^c^	3/7 (42.9%)	3 (2/1)	0	0	4 (2/2)
						
Non-UC						
Normal	7	0/7 (0%)	5 (5/0)	2 (2/0)	0	0
Inv. Ca	13^c^	5/13 (38.5%)	2 (1/1)	2 (2/0)	1 (1/0)	8 (4/4)

a Numbers of genetically analysed crypts are shown.

b p53 protein accumulations are classified into four categories: −=no positive staining cells; −/+=scattered staining in the crypt (REG, LGD, HGD) or positive for less than 25% (invasive carcinoma); +=positive in the lower half of the crypt (REG, LGD, HGD) or positive for 25–75% (invasive carcinoma); ++=positive in the whole crypt (REG, LGD, HGD) or positive for more than 75% (invasive carcinoma). Numbers in parentheses show wild-type crypts (lesions) *vs* mutant type crypts (lesions).

c In invasive carcinoma from UC and non-UC, the number shows the analysed lesions.

REG=regenerative epithelium; LGD=low-grade dysplasia; HGD=high-grade dysplasia; UC=ulcerative colitis; Inv. Ca.=invasive carcinoma.

The strategy of the combination of microdissection–PCR-direct sequencing and IHC is schematically illustrated in Figure 1A. Examples of the microdissection and sequencing data are shown in Figure 1B and C. Both p53 gene mutations and protein accumulation were analysed in 19 REG, 41 LGD, five HGD, seven UC-associated invasive carcinoma crypts or lesions. The former were found by PCR–direct sequencing in 42.1, 38.1, 100 and 42.9% of the cases, respectively. p53 protein accumulation was classified into four categories on the basis of IHC findings (see the legend of Table 1). Of the REG crypts, 73.7% (14 crypts out of 19) were negative for p53 accumulation and 26.3% (five crypts) were positive (+/−, + or ++). For LGD, the figures were 39.0% (16 out of 41) and 61.0%; for HGD, 20% (one out of five) and 80%; and for invasive carcinomas from UC, 42.9% (three lesions out of seven) and 57.1% (four lesions). Both kinds of discrepancies between p53 overexpression and gene mutations were apparent: (1) p53 overexpression (+)/gene mutation (−), (2) p53 overexpression (−)/gene mutation (+). In REG, three crypts showed the former (1) (15.8%) and six the latter (2) (31.5%), out of a total of 19 crypts. Likewise, for LGD, there were 19 (46.3%) and eight (19.5%) in 41 crypts; in HGD, only one crypt demonstrated a type 2 (20%) mismatch out of five crypts; in UC-associated invasive carcinomas, the figures were two (28.6%) and one (14.3%); and in non-UC-associated cancers, seven (53.8%) and one (7.7%).

### Immunohistochemistry of p53-related gene products (Figures 2, 3, 4 and 5)

**Figure 2:**
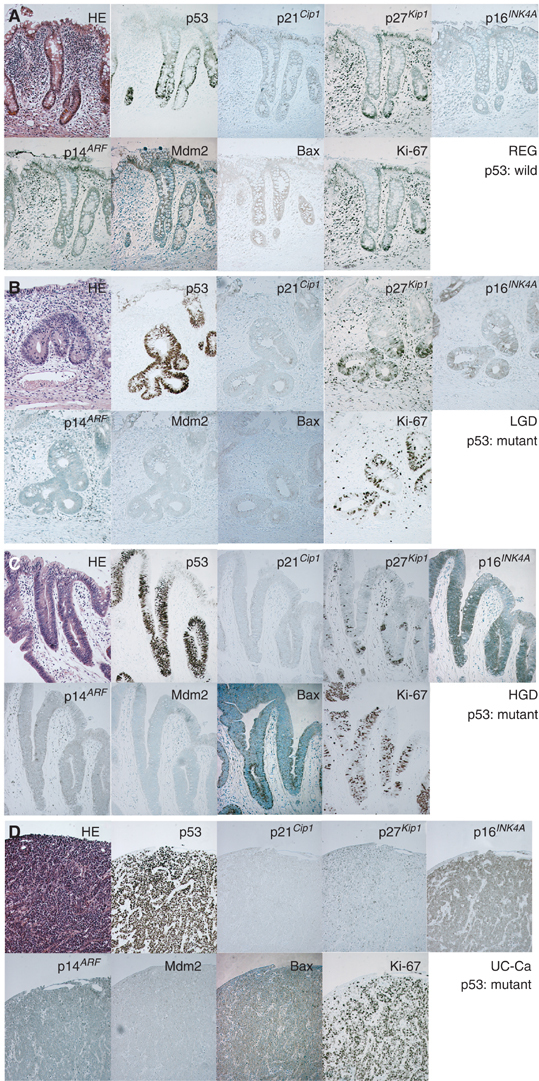
Morphology and immunohistochemical findings for UC-associated lesions. (**A**–**D**) HE, p53, p21^*Cip1*^, p21^*Kip1*^, p16^*INK4A*^, p14^*ARF*^, Mdm2, Bax and Ki-67 staining in semiserial sections (**A**: REG with wild p53 gene, **B**: LGD with mutant p53 gene, **C**: HGD with mutant p53 gene, **D**: invasive carcinoma with mutant p53 gene).

**Figure 3:**
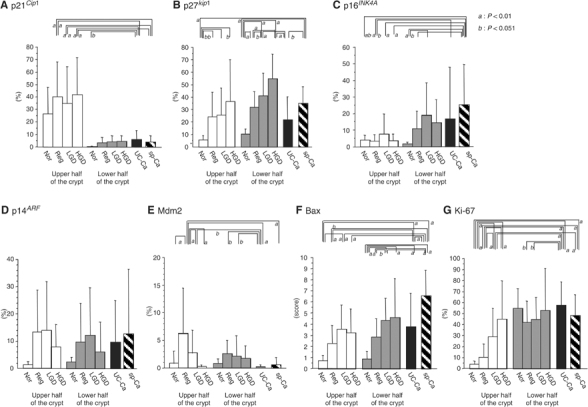
Immunopositivity for p53-related molecules in UC-associated lesions. Percentages or scores for immunopositivity are shown separately for the upper (open bars) and lower (gray bars) halves of the crypts. Data for UC-associated invasive carcinomas (UC-Ca) are shown in black bars and sporadic invasive carcinomas (sp-Ca) in hatched bars. Vertical lines show 1 s.d. **A**: p21^*Cip1*^, **B**: p27^*Kip1*^, **C**: p16^*INK4A*^, **D**: p14^*ARF*^, **E**: Mdm2, **F**: Bax and **G**: Ki-67. (*a*) *P*<0.01; (*b*) *P*<0.05 by Fisher's PLSD test.

**Figure 4:**
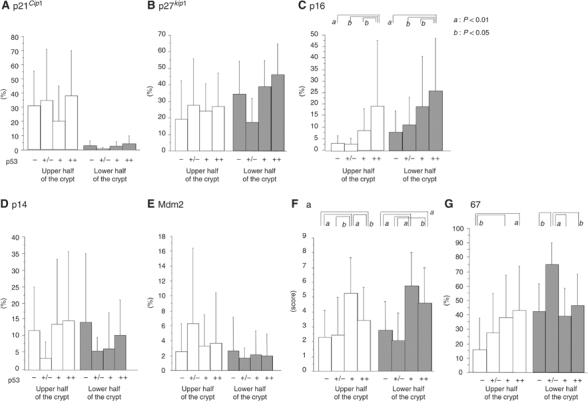
Immunopositivity of p53-related molecules compared to p53 overexpression in UC-associated dysplastic lesions and regenerative crypts. Percentages or scores for immunopositivity are shown as in Figure 2. **A**: p21^*Cip1*^, **B**: p27^*Kip1*^, **C**: p16^*INK4A*^, **D**: p14^*ARF*^, **E**: Mdm2, **F**: Bax and **G**: Ki-67. Degrees of p53 overexpression are shown as −, +/−, + and ++, as described in Materials and methods. (*a*) *P*<0.01; (*b*) *P*<0.05 by Fisher's PLSD test.

**Figure 5:**
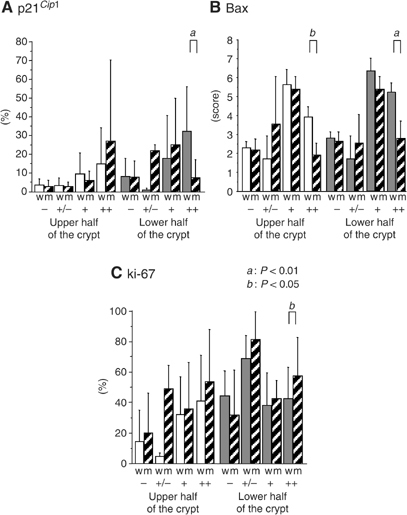
Immunopositivity of p16^*INK4A*^, Bax and Ki-67 compared to p53 gene mutation and overexpression in UC-associated dysplastic lesions and regenerative mucosa. Percentages or scores for immunopositivity are shown as in Figure 2. Open bars are for p53-wild type (w) and hatched bars for p53 mutated crypts (m). **A**: p16^*INK4A*^, **B**: Bax and **C**: Ki-67. Degrees of p53 overexpression are shown as −, +/−, + and ++, as described in Materials and methods. (*a*) *P*<0.01; (*b*) *P*<0.05 by Fisher's PLSD test.

Although p21^*Cip1*^ was expressed mainly in the upper half of the crypts in normal mucosa, REG, LGD and HGD, reduced expression was seen in UC-associated and non-UC-associated invasive carcinomas, the difference being significant (Figure 3A). p27^*Kip1*^ expression was greater in the lower than in the upper half of the crypt in normal, REG, LGD and HGD cases, and the expression level increased with progression of lesions (Figure 3B). In UC-associated invasive carcinomas, the expression was significantly reduced as compared to HGD and sporadic colon cancers. The expression of p21^*Cip1*^ and p27^*Kip1*^ demonstrated no significant differences in relation to the p53 expression in normal, REG, LGD, HGD mucosa (Figure 4A and B). Levels of p16^*INK4A*^ were significantly increased in invasive carcinomas (UC-associated and non-UC associated) compared to the both upper and lower half of the crypts of normal and REG mucosa (Figure 3C). In the lower half of the LGD crypts, expression was significantly higher than in normal mucosa. Limited to normal, REG, LGD, HGD mucosa, step-wise increase in p16^*INK4A*^ expression was seen with p53 overexpression (Figure 4C). Dividing into categories with or without genetic mutations of p53, mutant crypts with diffuse p53 overexpression had significantly decreased expression of p16 (Figure 5A).

With p14^*ARF*^, no significant variation was detected (Figure 3D). However, Mdm2 expression in regenerative mucosa was significantly higher than in normal, LGD, HGD crypts and in UC-associated and non-UC-associated invasive carcinoma lesions (Figure 3E). The expression of p14^*ARF*^ and Mdm2 demonstrated no significant relation with p53 levels (Figure 4D and E). With scoring for the intensity and areas of positive cells, there was a tendency for Bax expression to increase along with progress of histological features in both upper and lower crypts (Figure 3F). The score was significantly higher in LGD than in normal or REG mucosa (upper and lower crypt), in HGD than in normal mucosa (lower crypt), in UC-associated invasive carcinoma than in upper half of the normal or REG mucosa and in lower half of normal mucosa, and in sporadic invasive carcinomas than in UC-associated carcinomas. Bax expression was also significantly higher in p53 + or ++ crypts than in their p53− or +/− counterparts (Figure 4F). Such increase was not seen in p53 mutant crypts with diffuse p53 overexpression (p53 ++) (Figure 5B). Cell cycling, as indicated by Ki-67 expression, was increased along with the histological stage and the p53 expression level in upper crypts (Figures 3G and 4G). Additionally, p53 mutant crypts with diffuse p53 overexpression showed significantly higher Ki-67 labelling indices than wild-type crypts (Figure 5C). Compared to the lower halves of crypts of REG or LGD mucosa, cell cycling was increased in UC-associated invasive carcinomas.

### Bax and p16^*INK4A*^ expression by immunoblotting and quantitative PCR (Figure 6)

**Figure 6:**
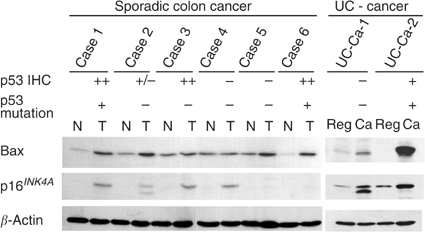
Immunoblotting of p16^*INK4A*^ and Bax in representative sporadic and UC-associated invasive carcinomas. Immunohistochemical expression of p16^*INK4A*^ and Bax was confirmed by immunoblotting. p53 IHC, p53 overexpression analysed immunohistochemically in identical lesions; p53 gene mutations determined by PCR–SSCP and DNA sequencing; *β*-actin, quantitative standard. Molecular weight marker (15 k) is shown with p16 bands.

By immunoblotting, increased expression levels of p16^*INK4A*^ and Bax were evident in UC-associated invasive carcinoma tissue as compared to UC-regenerative mucosa, correlating with the immunohistochemical results. Increase of Bax and p16^*INK4A*^ expression was also seen in sporadic invasive carcinomas (Bax, four out of six examined cases; p16^*INK4A*^, four out of six examined cases), although the two did not well match each other. p53 gene mutations in sporadic invasive carcinomas also did not coincide with Bax and p16^*INK4A*^ expression.

Threshold cycles (*C*_T_'s) for p16 and GAPDH of nontumour tissue of UC and UC-associated tumour were obtained by real-time quantitative PCR. From the formula described in Materials and methods, cDNA ratios of p16 (tumour *vs* normal tissue) and GAPDH were 1371 and 8.456. Under the assumption that the efficiency of reverse transcription was constant, the induction ratio of mRNA of p16 (tumour *vs* normal tissue) is estimated 162.2 after compensation for GAPDH induction.

## DISCUSSION

As confirmed by the present study, p53 gene mutations and p53 protein overexpression in malignant neoplasms are not necessarily concordant. In our previous report, although there was coincidence in eight out of 20 cases of sporadic colorectal carcinoma, mismatches were found in 11 cases (Yamashita *et al*, 2001). In these lesions, we carefully sequenced in forward and reverse directions to exclude technical errors to the best of our ability. The possibility that a mutation outside the coding region could have influenced the expression or function of p53 must also be borne in mind. However, so far we examined, in the present study, three (15.8%), 19 (46.3%) and 0, REG, LGD and HGD crypts of UC, respectively, demonstrated overexpression despite a lack of any mutation. In addition, six (31.5%), eight (19.5%) and one (20.0%), respectively, had mutations but no overexpression. There are possible explanations for this type of mismatch group. At least, as the epitope of DO-7 used in immunostaining is amino-terminus of p53, and the hot spot of p53 mutation is in the relatively carboxyl-terminus, most (but not all, perhaps) of the mutant p53 could be detected by DO-7. Although, the analysed crypt numbers for HGD were rather small, making conclusions difficult, mismatches were very prevalent in REG or LGD, early stages in UC-associated tumorigenesis.

Since it is of interest to ascertain whether the molecular mechanisms are dependent or independent of p53 or related molecules in tumorigenesis, we analysed negative cell cycle regulators, the cyclin-dependent kinase inhibitors p21^*Cip1*^, p27^*Kip1*^ and p16^*INK4A*^, the p53-related cell cycle regulators, p14^*ARF*^ and Mdm2, a proapoptosis molecule, Bax, and a proliferation marker, Ki-67, in the present study. Generally, p21 and p27 are expressed in inverse correlation to the progression of carcinomas, and p16 and p14 are known to be methylated in many carcinoma cases. Mdm2 degrades p53 and thus keeps the endogenous level of the protein low. Bax, the apoptosis-inducing factor, has a p53-responsive element in its promoter region and is induced by p53. In the present study, Mdm2 expression was significantly increased only in regenerative crypts compared to normal crypts, suggesting a positive role for p53 protein degradation. We here revealed that, in UC-associated lesions, p21 expression is significantly reduced in invasive carcinomas compared to normal mucosa, and that p27 expression tends to increase along with progression of dysplasia but is also significantly decreased in later stages. This result suggests that in dysplasia, p21 and p27 may provide a negative feedback against progression, but that this is lost with onset of malignancy.

Although we did not find a significant difference in p14 with progression from dysplasia to carcinoma, step-wise induction of p16 was seen. This is an unexpected result in light of the previous finding that p16 promoter methylation is frequent in UC-associated dysplasia or carcinomas (Hsieh *et al*, 1998). While the discrepancy remains to be clarified, increased p16 expression at the protein level was also confirmed biochemically in UC-associated carcinomas and sporadic carcinomas of colorectum in the present study. Since the induction was also confirmed by real time-PCR at mRNA level, the increased p16 expression suggested to be due to increased transcription, and not to protein stabilisation. Interestingly, increase of p16 expression in dysplasia was accompanied by positive p53 overexpression of the wild-type gene, whereas the expression level in dysplasia of mutant type was significantly lower. Likewise, the Bax expression in p53-overexpressed crypts was increased along with progression of dysplasia, but only in lesions without any mutation. Since Bax is a downstream effector of p53, this is reasonable. There are two types of diffuse overexpression of p53, with a wild-type or mutant gene status. The former can directly induce Bax, and may indirectly induce p16 as part of the negative feedback system, but the latter cannot. Therefore, in tumours with mutated p53, failure of the p53 regulation system might be a key event for tumorigenesis. On the other hand, without any mutation, the p53 system might be intact and dysfunction of other regulating pathways is involved. Recently deregulation of the *β*-catenin and Wnt signalling pathway has been reported to induce cell proliferation when p14^*ARF*^ function is lacking (Damalas *et al*, 2001), and a possible relation of dysfunction of *β*-catenin with UC-associated tumours has been reported (Walsh *et al*, 1999; Mikami *et al*, 2000; Aust *et al*, 2001; Haq *et al*, 2001). Further study of *β*-catenin and Wnt signalling pathways, concurrently with genetic analysis of p53 should provide clues regarding this question.
